# Sulfonamides identified as plant immune-priming compounds in high-throughput chemical screening increase disease resistance in *Arabidopsis thaliana*

**DOI:** 10.3389/fpls.2012.00245

**Published:** 2012-10-31

**Authors:** Yoshiteru Noutoshi, Mika Ikeda, Tamio Saito, Hiroyuki Osada, Ken Shirasu

**Affiliations:** ^1^Research Core for Interdisciplinary Sciences, Okayama UniversityOkayama, Japan; ^2^Chemical Biology Core Facility, RIKEN Advanced Science InstituteSaitama, Japan; ^3^RIKEN Plant Science CenterYokohama, Japan

**Keywords:** *Arabidopsis thaliana*, *Pseudomonas syringae*, plant activator, immune-priming, hypersensitive cell death, sulfonamide, high-throughput chemical screening

## Abstract

Plant activators are agrochemicals that protect crops from diseases by activating the plant immune system. To isolate lead compounds for use as practical plant activators, we screened two different chemical libraries composed of various bioactive substances by using an established screening procedure that can selectively identify immune-priming compounds. We identified and characterized a group of sulfonamide compounds – sulfameter, sulfamethoxypyridazine, sulfabenzamide, and sulfachloropyridazine – among the various isolated candidate molecules. These sulfonamide compounds enhanced the avirulent *Pseudomonas*-induced cell death of *Arabidopsis* suspension cell cultures and increased disease resistance in *Arabidopsis* plants against both avirulent and virulent strains of the bacterium. These compounds did not prevent the growth of pathogenic bacteria in minimal liquid media at 200 μM. They also did not induce the expression of defense-related genes in *Arabidopsis* seedlings, at least not at 24 and 48 h after treatment, suggesting that they do not act as salicylic acid analogs. In addition, although sulfonamides are known to be folate biosynthesis inhibitors, the application of folate did not restore the potentiation effects of the sulfonamides on pathogen-induced cell death. Our data suggest that sulfonamides potentiate *Arabidopsis* disease resistance by their novel chemical properties.

## INTRODUCTION

Plants have an innate immune system that requires the phytohormone salicylic acid (SA) for disease resistance against pathogens, including fungi, bacteria, and viruses (Vlot et al., 2009). Upon pathogen infection, plants recognize pathogen-derived effector molecules by using cytosolic immune sensors and activate SA biosynthesis to mount disease resistance responses (Dangl and Jones, 2001). This often results in a hypersensitive response (HR) that leads to the rapid induction of programed cell death at sites of infection (Mur et al., 2008).

Plant activators are agrochemicals that confer disease resistance to crops by activating plant immune systems. Due to their nature, plant activators work on host plants and not on pathogens; therefore, no drug-resistant microbes have emerged as a result of their longstanding use in the fields. This durability is one of the advantages of plant activators. Probenazole (3-prop-2-enoxy-1,2-benzothiazole 1,1-dioxide, Oryzemate^®^) is a practically used plant activator that was incidentally discovered 35 years earlier through fungicide screening (Watanabe et al., 1977). Probenazole protects rice against blast fungus and bacterial blight without exerting antimicrobial activities. It is used prophylactically and can, as a result, decrease not only yield loss but also the usage of commonly used pesticides and farmer’s workloads. Probenazole does not act like an SA analog and it might induce a primed state in plants for defense responses. Defense priming is known as a unique physiological state that can be induced by treatment with some natural or synthetic compounds and by wounding. Primed plants show a more rapid and robust activation of defense responses against subsequent challenges by microbes, insects, or abiotic stress (Conrath, 2011). The chemical effects of probenazole have been found to be exerted through accumulation of SA in *Arabidopsis*, tobacco, and rice (Yoshioka et al., 2001; Nakashita et al., 2002; Iwai et al., 2007; Umemura et al., 2009). However, the precise mode of action of probenazole has not yet been identified.

The success of probenazole promoted the hunt for plant activators from synthetic chemicals or biotic sources by using various approaches. A number of bioactive molecules were subsequently isolated, including BTH (*S*-methyl 1,2,3-benzothiadiazole-7-carbothioate; Schreiber and Desveaux, 2008). BTH (Actigard^®^, formerly registered as BION^®^) is the synthetic analog of SA and is actually used in the field as a treatment for certain diseases of cole crops, leafy vegetables, tomatoes, and tobacco (Gorlach et al., 1996; Lawton et al., 1996). INA (2,6-dichloroisonicotinic acid) is also a potent SA analog but it is not used practically due to its phototoxic effects (Ward et al., 1991). This is also the case with other commercially available plant activators that induce a strong defense response, which is often associated with severe growth suppression, when they are overused (Shirano et al., 2002; Zhang et al., 2003; Noutoshi et al., 2005, 2006; Yang et al., 2012).

Tiadinil (V-get^®^) and isotianil (Stout^®^) are recently registered plant activators that mainly target the rice cultivation market (Himmler, 2004; Tsubata et al., 2006). Their mechanism of action is likely to be different from that of probenazole. Tiadinil has been found to be metabolized in plants to form SV-03, which induces the expression of defense genes without SA accumulation (Yasuda et al., 2006). Judging from their molecular structures, these compounds could have been developed by modifying pre-existing commercial compounds.

Like human drug discovery, large-scale screening of a broad range of compounds is useful in identifying lead compounds that could be novel plant activators applicable to various crops. However, evaluation of chemical effects on plant disease resistance by using actual plants and pathogens requires a large quantity of chemicals, thus restricting the range of chemicals that can be tested. Recently, a bulk bioproduction technique was developed for rare monosaccharides, which helped identify D-allose and D-psicose as disease resistance inducers in rice (Kano et al., 2010, 2011). Establishment of small-scale chemical screening procedures using young *Arabidopsis* seedlings has enabled the screening and identification of bioactive plant activators from a chemical library that included a large number of various small organic molecules (Serrano et al., 2007; Schreiber et al., 2008; Knoth et al., 2009). However, simple inducers of defense responses such as chemical elicitors or SA analogs could be associated with phytotoxicity. To avoid such unfavorable side effects, the candidate lead compounds are expected to potentiate but not constitutively induce plant immune responses.

In a previous study, we established a high-throughput assay system that could quantitatively monitor cell death in *Arabidopsis* suspension-cultured cells (Noutoshi et al., 2012a). Using this method, we evaluated the effects of various chemical compounds on avirulent pathogenic *Pseudomonas*-induced cell death. To selectively identify compounds that enhance but not induce cell death, cell death inducers were eliminated by evaluating their chemical effects on cell viability in the absence of a pathogen, in parallel. Thus, several plant immune-priming compounds could be identified from a chemical library of 10,000 diverse small molecules (Noutoshi et al., 2012a).

In this study, we screened two chemical libraries composed of bioactive molecules and natural products and identified four sulfonamides as plant immune-priming compounds. These chemicals potentiated pathogen-induced cell death and increased disease resistance in *Arabidopsis* plants.

## MATERIALS AND METHODS

### CHEMICALS

A commercially available chemical library of 1,000 medical drugs, 500 natural products with unknown biological properties, and 420 non-drug bioactive compounds was purchased from a chemical supplier (The Spectrum Collection; 10 mM in DMSO; MicroSource Discovery Systems Inc., Gaylordsville, CT, USA). Note that the 11 compounds in this library assigned as prohibited imports by customs regulations and were not tested. A publicly available chemical library of 768 chemicals composed of bioactive molecules and natural products was obtained from the RIKEN Natural Products Depository (NPDepo800; 10 mg/mL in DMSO; RIKEN ASI, Saitama, Japan; Osada, 2010). Sulfamethoxypyridazine (S0591) and sulfabenzamide (S0582) were purchased from Tokyo Chemical Industry (Tokyo, Japan). Sulfameter (sulfamethoxydiazine, S0383), sulfachloropyridazine (S9882), and sodium salicylate (S3007) were from Sigma-Aldrich (St. Louis, MO, USA).

### PLANT MATERIALS AND GROWTH CONDITIONS

*Arabidopsis* suspension-cultured cells were grown in liquid media containing MS with 3% sucrose supplemented with 0.5 mg/L MES (pH 5.7), 0.5 mg/L naphthaleneacetic acid, and 0.05 mg/L 6-benzyl amino purine under long-day conditions (Menges and Murray, 2002; Maor et al., 2007). For gene expression analysis, *A. thaliana* ecotype Columbia was grown on half-MS agar medium (1% sucrose) at 22°C under long-day conditions (16-h light/8-h dark cycles). To assay for disease resistance, plants were grown hydroponically at 22°C under short-day conditions (8-h light/16-h dark cycles). For growth assays, *Arabidopsis* seeds were sterilized and stored at 4°C for 4 days to break dormancy. The seeds were then dispensed into 96-well plates and grown in half-MS liquid medium supplemented with 1% sucrose with 1–50 μM of the chemicals. The plates were subsequently incubated at 22°C under long-day conditions.

### ASSAY FOR CHEMICAL EFFECT ON THE CELL DEATH OF *Arabidopsis* SUSPENSION CULTURES

The method used has been previously described (Noutoshi et al., 2012a). *Arabidopsis* suspension cells were dispensed into each well of 96-well plates with deep wells, and 10–100 μM of the chemicals were applied into two wells. DMSO and SA were used as negative and positive controls, respectively. After 1-h incubation, *Pseudomonas syringae* pv. *tomato* DC3000 (*Pst*) *avrRpm1* (final concentration; OD = 0.2 in MS medium without hormones) was applied into one of the duplicated wells. As a mock, MS medium without bacteria was used. After cocultivation on a shaker for 21 h under long-day conditions at 22°C, cells were stained with 1% Evans blue dye. The cells were then washed four times with 1 mL of water and any incorporated dye was extracted with 400 μL of an elution solution (50% methanol, 1% SDS). The absorbance at 595 nm was measured by a microplate reader. Cell viability was calculated as a relative value with the negative control considered as 100.

### PLANT CHEMICAL TREATMENTS AND RNA EXPERIMENTS

For RNA experiments with pathogen-infected samples, *Arabidopsis* WT seedlings (Columbia ecotype) grown on half-MS agar plates (1% sucrose) for 1 week under short-day conditions, that is, 8 h light/16 h dark, were transferred onto rockwool and hydroponically cultivated at 22°C. After 3 weeks, plants were transferred into small pots supplemented with or without 100 μM solution of each chemical for 3 days before spray-inoculation with bacteria. Rosetta leaves were collected in 2 mL tubes and frozen in liquid nitrogen. For experiments with the chemical-treated samples without the pathogen, *Arabidopsis* seedlings (Col) grown in half-MS medium (1% sucrose) for 2 weeks were soaked in liquid half-MS medium (1% sucrose) supplemented with 100 μM of the chemicals. The plants were incubated for 24 or 48 h at 22°C, and the seedlings were collected in 2 mL tubes and frozen in liquid nitrogen.

The samples were crushed with four zirconia balls (ø, 2 mm) by using a Shake Master Neo (BMS, Tokyo, Japan). Total RNA was extracted using the PureLink Micro-to-Midi Total RNA Purification System with the on-column DNase treatment procedure (Invitrogen, Carlsbad, CA, USA), and RNA concentrations and purity were measured with a spectrometer at 260 and 260/280 nm, respectively (BioPhotometer Plus, Eppendorf, Germany). cDNA was synthesized with a PrimeScript RT reagent kit and with gDNA Eraser (Perfect Real Time; Takara, Shiga, Japan). Quantitative reverse transcriptase polymerase chain reaction (qRT-PCR) amplifications were performed in 96-well plates with a LightCycler^®^ 480 real-time thermocycler (Roche Diagnostics, Basel, Switzerland) using a KAPA SYBR Fast qPCR Kit (KapaBiosystems, Woburn, MA, USA). Quantification of the target transcript was carried out using the LightCycler 480 internal software Absolute Quantification 2nd Derivative Max and normalized to *Actin2*. The primers were 5′-CCGCTCTTTCTTTCCAAGC-3′ and 5′-CCGGTACCATTGTCACACAC-3′ for *Actin2*, 5′-TGATCCTCGTGGGAATTATGT-3′ and 5′-TGCATGATCACATCATTACTTCAT-3′ for *pathogenesis-related 1* (*PR1*), and 5′-CTTAGCCTCACCACCAATGTTG-3′ and 5′-TCCCGTAGCATACTCCGATTTG-3′ for *At3g57260*.

### BACTERIAL GROWTH ASSAY

*Pseudomonas syringae* pv. *tomato* DC3000 was precultured in M9 minimal medium (Na_2_HPO_4_, 7 mg/mL; KH_2_PO_4_, 3 mg/mL; NaCl, 0.5 mg/mL; NH_4_Cl, 1 mg/mL; thiamin, 5 μg/mL; MgSO_4_, 0.12 mg/mL; glucose, 4 mg/mL) with kanamycin (50 μg/mL) at 28°C and was inoculated into fresh M9 medium to a final optical density of 0.02 at 600 nm (OD_600_). The medium was supplemented with sulfonamide (200 μM) or hygromycin (100 μg/mL) and the growth of bacteria was monitored by measuring the absorbance at 600 nm (OD_600_).

### ENZYME ASSAY FOR SA GLUCOSYLTRANSFERASE

Enzyme assays for SA glucosyltransferase (SAGT) activity were performed as described previously (Noutoshi et al., 2012a). Each assay (40 μL) mixture contained 2.5 μg/mL of UGT74F1, 0.28 mM SA, 0.33 mM UDP-glucose, 14 mM 2-mercaptoethanol in 50 mM MES, pH 8.0. Then, 100 μM each of sulfonamide or imprimatin A2, one of the immune-priming compounds which we identified in the previous study (Noutoshi et al., 2012a), was added to the reaction mixes and incubated at 30°C for 30 min before the reaction was stopped by the addition of 8 μL of 50% (v/v) trichloroacetic acid. SAG was detected with HPLC by using a fluorescence detector (excitation, 295 nm; emission, 370 nm).

### ASSAY FOR DISEASE RESISTANCE IN PLANTS

Inoculation and measurement of bacterial growth in plants were performed as described previously (Weigel and Glazebrook, 2002).* Arabidopsis* seedlings grown on MS agar plates for 1 week under short-day conditions were transferred onto rockwool and hydroponically cultivated at 22°C. After 3 weeks, plants with rockwool were transferred into small pots and supplied with water containing various chemicals (100 μM) or SA (50 μM). After 3 days, freshly grown bacteria of both *Pst* and *Pst-avrRpm1* strains were resuspended in 10 mM MgCl_2_ (OD_600_ = 0.002) and were used to inoculate leaves with a needle-less syringe. Then, 50 μM leaf disks were then punched out with a sterile cork-borer at 0 and 3 days after inoculation. Samples were collected in a 2-mL tube and ground with zirconia balls (ø, 2 mm) with 500 μL of 10 mM MgCl_2_. Dilution series of each sample were spread on agar media containing kanamycin and rifampicin, and the number of bacteria inside the leaves was calculated.

## RESULTS

### ISOLATION OF FOUR SULFONAMIDES AS PLANT IMMUNE-PRIMING COMPOUNDS

**FIGURE 1:**
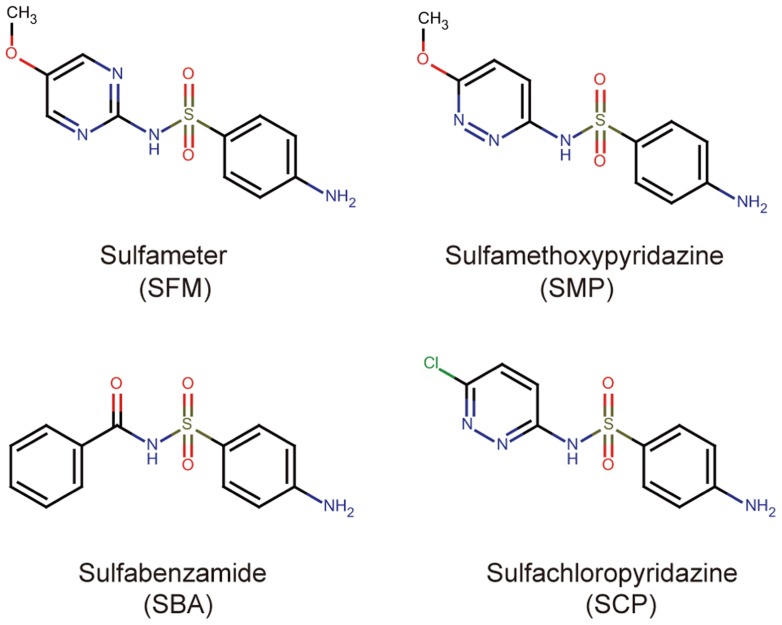
**Chemical structures of the sulfonamide compounds isolated from the chemical screening**.

By using the previously established chemical screening system (Noutoshi et al., 2012a), we tried to identify compounds that enhance but not induce the death of *Arabidopsis* suspension-cultured cells triggered by infection with avirulent pathogenic *Pst*-*avrRpm1*. In this study, we screened two different chemical libraries. One is a commercially available library that contains 2,000 small organic molecules and is composed of known pharmaceutical drugs, experimental bioactives, and natural products supplied by MicroSource Discovery Systems Inc. The other is a publicly available NPDepo library provided by RIKEN, which consists of 768 bioactive molecules and natural products (Osada, 2010). After three replications of the screening procedures, the candidate chemicals were reassessed for their concentration-dependent ability to promote immune responses. The various compounds that enhanced pathogen-induced cell death reproducibly were isolated. These candidate molecules were classified into several groups based on both bioactivities and molecular structures. Here, we characterized one of these groups, which consists of four different sulfonamide compounds: sulfameter (SFM), sulfamethoxypyridazine (SMP), sulfabenzamide (SBA), and sulfachloropyridazine (SCP; **Figure 1**). SFM and SMP were isolated from the MicroSource library, and SBA and SCP were from the NPDepo library (**Figure 1**).

**FIGURE 2:**
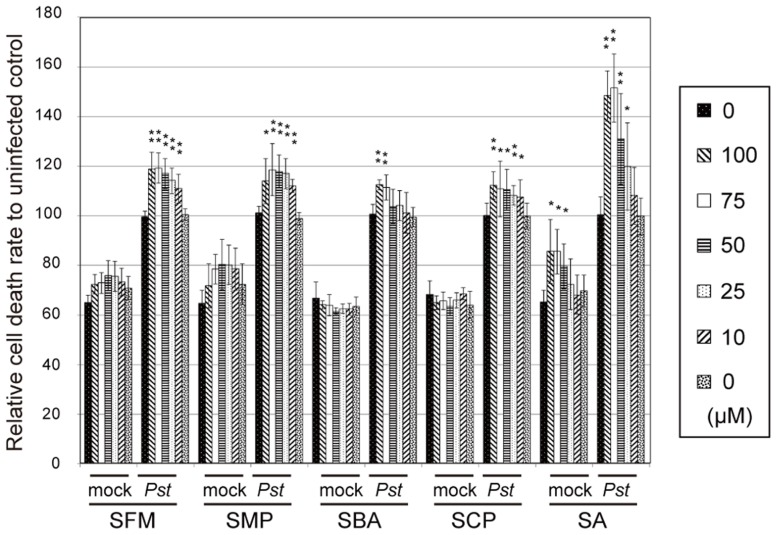
**The effects of the isolated sulfonamides on cell death in *Arabidopsis* suspension cultures upon challenge with pathogenic bacteria**. The compounds were incubated with *Arabidopsis* suspension-cultured cells with or without the avirulent bacterial pathogen *Pst-avrRpm1*, and the degree of cell death was measured using Evans blue dye. Each cell death rate is shown as a value relative to the mean for the mock pathogen-treated group. SA was used as a positive control. The error bars represent mean ± SE values of four independent replicates. **P < 0.01; two-tailed Student’s *t* test with *post hoc* Bonferroni’s correction.

These sulfonamide compounds were treated with *Arabidopsis* suspension cells at varied concentrations, as shown in **Figure 2**. The rates of cell death were then quantitatively measured after cocultivation with *Pst-avrRpm1* for 21 h. We found that the compounds upregulated the cell death induced by *Pst-avrRpm1* in a concentration-dependent manner (**Figure 2**). They did not exhibit apparent toxicity with respect to cell viability in the absence of pathogen challenge. The cell death-enhancement activities of these compounds were weaker than those of SA, which functions as an endogenous potentiator for pathogen-induced cell death (Shirasu et al., 1997).

### EFFECT OF THE SULFONAMIDES ON DISEASE RESISTANCE IN *Arabidopsis* PLANTS

**FIGURE 3:**
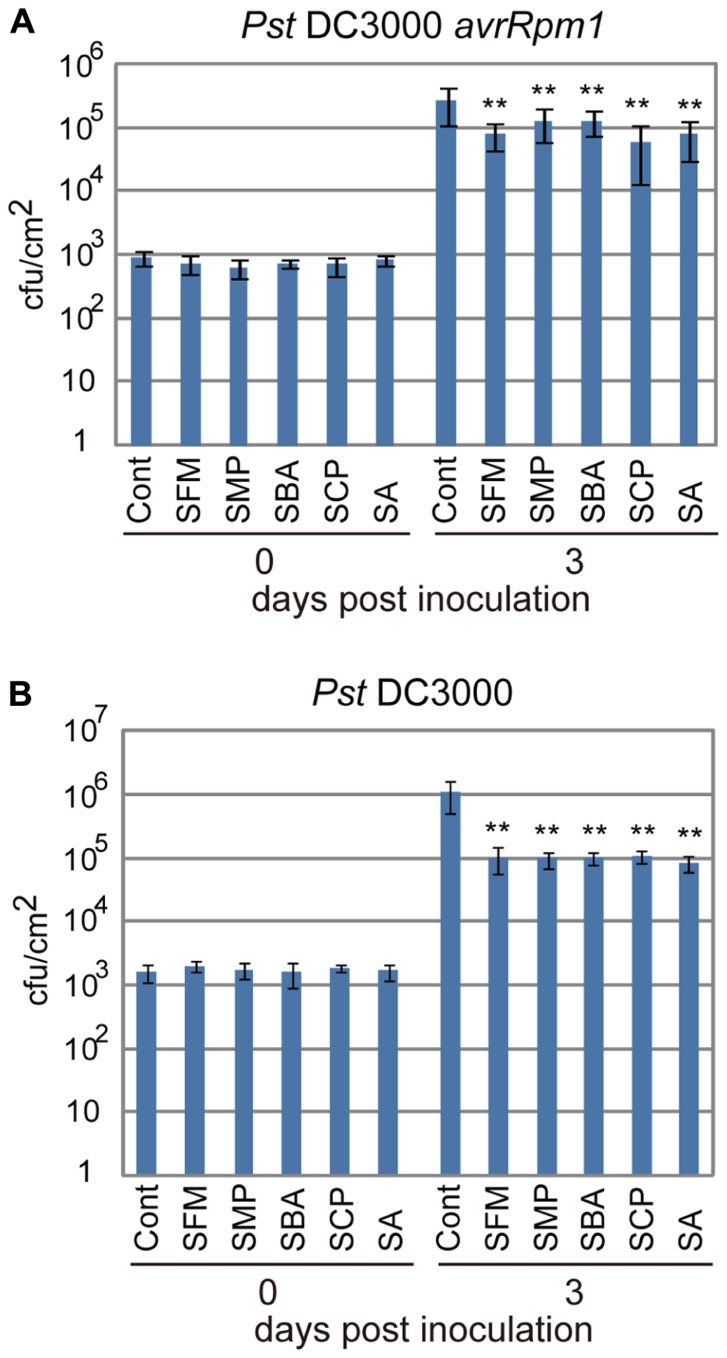
**Disease resistance of *Arabidopsis* plants treated with the sulfonamides**. *Arabidopsis* plants grown on rockwool for 3 weeks under short-day conditions were drenched for 3 days with water supplemented with 100 μM of each compound. As a positive control, 50 μM of sodium salicylate (SA) was used. Then, the avirulent *Pst-avrRpm1* **(A)** and the virulent *Pst* **(B)** were inoculated into leaves by infiltration with needle-less syringes (OD_600_ = 0.002 in 10 mM MgCl_2_) and bacterial counts inside leaves were counted at 0 and 3 days after inoculation. The error bars represent mean ± SE values (*n* = 7). *P < 0.01; two-tailed Student’s *t* test. These data are representative of two independent replicates with similar results.

**FIGURE 4:**
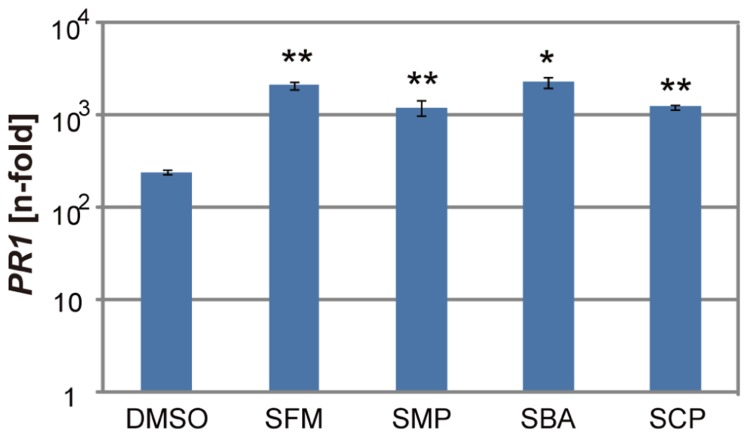
**Expression of the *PR1* gene in the sulfonamide-treated *Arabidopsis* plants during pathogen infection**. Hydroponically grown *Arabidopsis* seedlings were drenched with water containing 100 μM of each chemical for 3 days before inoculation of *Pst-avrRpm1* with spraying. The mRNA levels of *PR1* were determined by qRT-PCR with cDNA prepared from samples at 24 h after inoculation. The expression values were normalized using *Actin2* as an internal standard. The data was represented as relative values to the uninoculated samples with DMSO treatment. The error bars represent mean ± SE values of three independent replicates. *P < 0.01; two-tailed Student’s *t *test.

To examine if these sulfonamide compounds can function in plants, they were applied to *Arabidopsis* seedlings and their disease resistance was analyzed. The roots of hydroponically grown *Arabidopsis* seedlings were drenched in water solutions supplemented with each of the sulfonamides at the concentration of 100 μM; the positive and negative controls were 50 μM of SA and DMSO, respectively. After incubation for 3 days, each strain of virulent and avirulent bacteria of *Pst* was inoculated into leaves with a needle-less syringe and the numbers of bacteria inside leaves were counted at the 3 days after inoculation. Compared with the control treatment, all the sulfonamide compounds significantly decreased the growth of the avirulent bacteria (**Figure 3A**). These data indicate that the sulfonamides function *in planta* and increase the disease resistance of *Arabidopsis* seedlings. In addition to disease resistance against the avirulent bacterial strain used in the screening, they also conferred disease resistance against the virulent *Pst* strain (**Figure 3B**).The degree of resistance conferred by these molecules was similar to that conferred by 50 μM of SA. We also examined the expression level of *PR1*, a marker of SA-dependent defense responses, in the chemical-treated *Arabidopsis* plants during infection by *Pst-avrRpm1*. Consistent with the increases in disease resistance, enhanced *PR1* gene expression was observed in plants treated with the sulfonamides (**Figure 4**).

### ACTIVITY OF THE SULFONAMIDES FOR INDUCTION OF DEFENSE GENES

**FIGURE 5:**
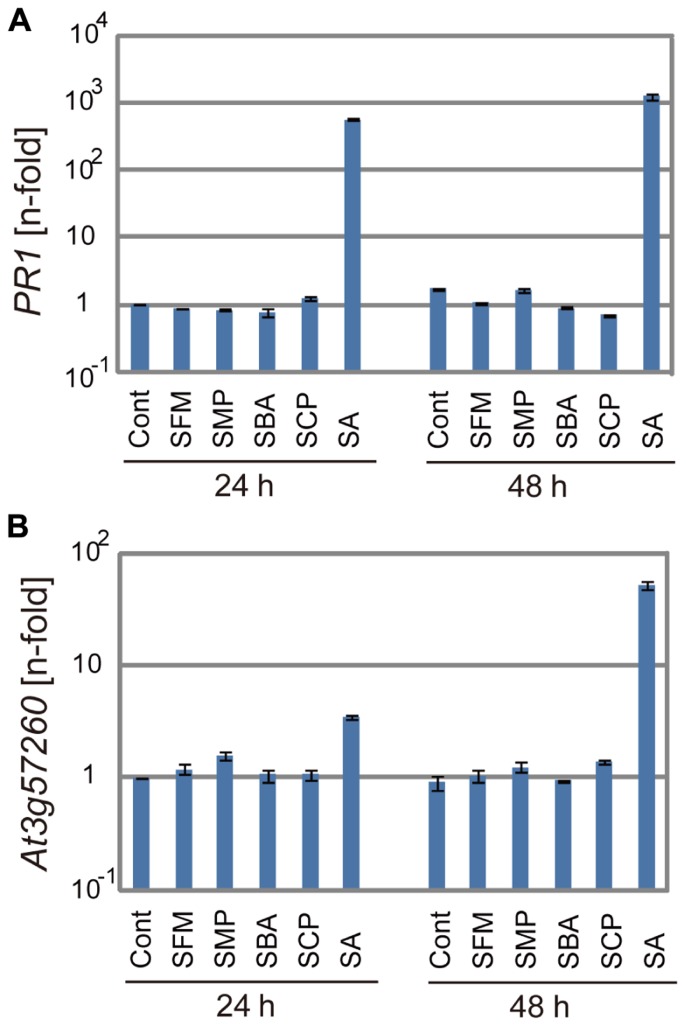
**The expression of defense genes after application of the sulfonamides**. The mRNA levels of *PR1* **(A)** and *At3g57260* **(B)** were determined by qRT-PCR with cDNA prepared from 10-day-old seedlings soaked for 24 or 48 h in liquid media containing 100 μM of each chemical. The expression values of the individual genes were normalized using *Actin2* as an internal standard. The data represent relative values with respect to the control. The bars represent the mean ± SE values of three independent replicates.

We examined if these isolated sulfonamide compounds induced defense genes like SA. *Arabidopsis* seedlings were treated with 100 μM of these compounds for 24 or 48 h, and the transcription of two defense genes, *PR* and SA-inducible gene *At3g57260*, was analyzed by real-time qRT-PCR (Knoth et al., 2009). As shown in **Figure 5**, the sulfonamides did not induce the expression of the defense genes, in contrast to SA, which effectively upregulated their mRNA transcription at various time points. These results indicate that the sulfonamide compounds do not behave like analogs of SA. However, it is still possible that the sulfonamides induce defense genes at other timepoints within 24 h.

### EFFECT OF THE SULFONAMIDES ON SA METABOLISM

**FIGURE 6:**
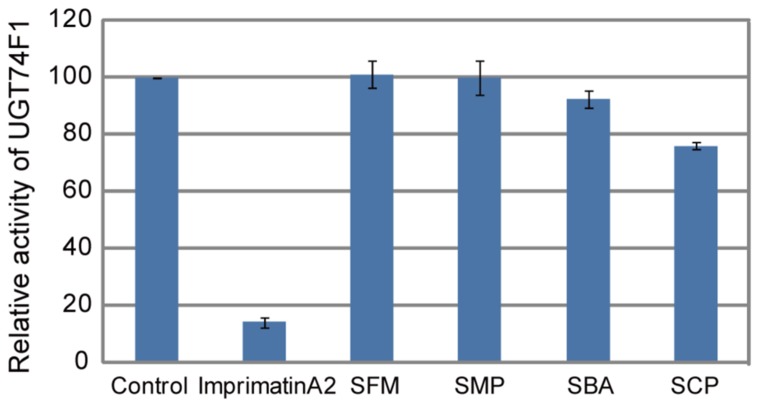
**Effect of the sulfonamides on the enzymatic activities of the SAGT.** The levels of SA-β-D-glucoside (SAG) in the *in vitro* enzymatic reaction using affinity-purified histidine-tagged recombinant *Arabidopsis* UGT74F1 protein expressed in *E. coli* were measured by HPLC. The sulfonamides and imprimatin A2 were provided at a concentration of 100 μM. The data are shown as values relative to the DMSO control. The bars represent the mean ± SE values of three independent replicates.

In our previous study, we found that perturbation of SA glucosylation, a major metabolic pathway during defense responses, is one of the modes of action of plant immune-priming agents (Dempsey et al., 2011; Noutoshi et al., 2012a). We isolated the immune-priming compounds imprimatin A and B from a chemical library of 10,000 small molecules by using our established screening procedure and found that they target known SAGTs and also novel SAGTs (Noutoshi et al., 2012a,b). Therefore, we examined whether the isolated sulfonamides inhibited the enzymatic activity of SAGTs. Each compound was added to the *in vitro* reaction mixture of one of the *Arabidopsis* SAGTs, UGT74F1, and their effects were evaluated. As compared with the imprimatin A2 positive control, which clearly inhibited SAGT activity, the sulfonamides did not prevent SAGT enzymatic activity at 100 μM, although SBA and SCP showed subtle inhibitory effects (**Figure 6**).

### EFFECTS OF THE KNOWN PROPERTY OF THE SULFONAMIDES ON IMMUNE-PRIMING

**FIGURE 7:**
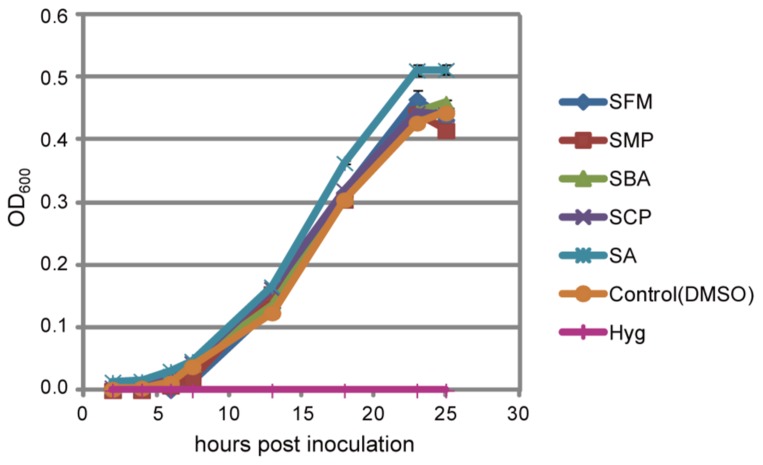
**Effect of the sulfonamides on bacterial growth**. *Pst* was cultured in liquid minimal medium supplemented with 200 μM of the indicated chemicals or 100 μg/mL hygromycin, and bacterial growth was monitored as the optical density of bacteria at 600 nm at the indicated times after inoculation. The bars represent the mean ± SE values of three independent replicates.

Sulfonamides are known to function as antibiotics that inhibit folate biosynthesis by mimicking *p*-amino benzoate (PABA), a precursor of folate; however, their effective concentrations for bacterial growth suppression are varied (Brown, 1962; Yun et al., 2012). We tested if *Pst* growth is prevented in the presence of these isolated sulfonamides at effective concentration ranges for the potentiation of pathogen-induced cell death of *Arabidopsis* cell suspensions. The sulfonamides did not interrupt bacterial growth at least until a concentration of 200 μM, although we did not measure the chemical concentration in the leaves of the sulfonamide-treated *Arabidopsis* plants (**Figure 7**).

**FIGURE 8:**
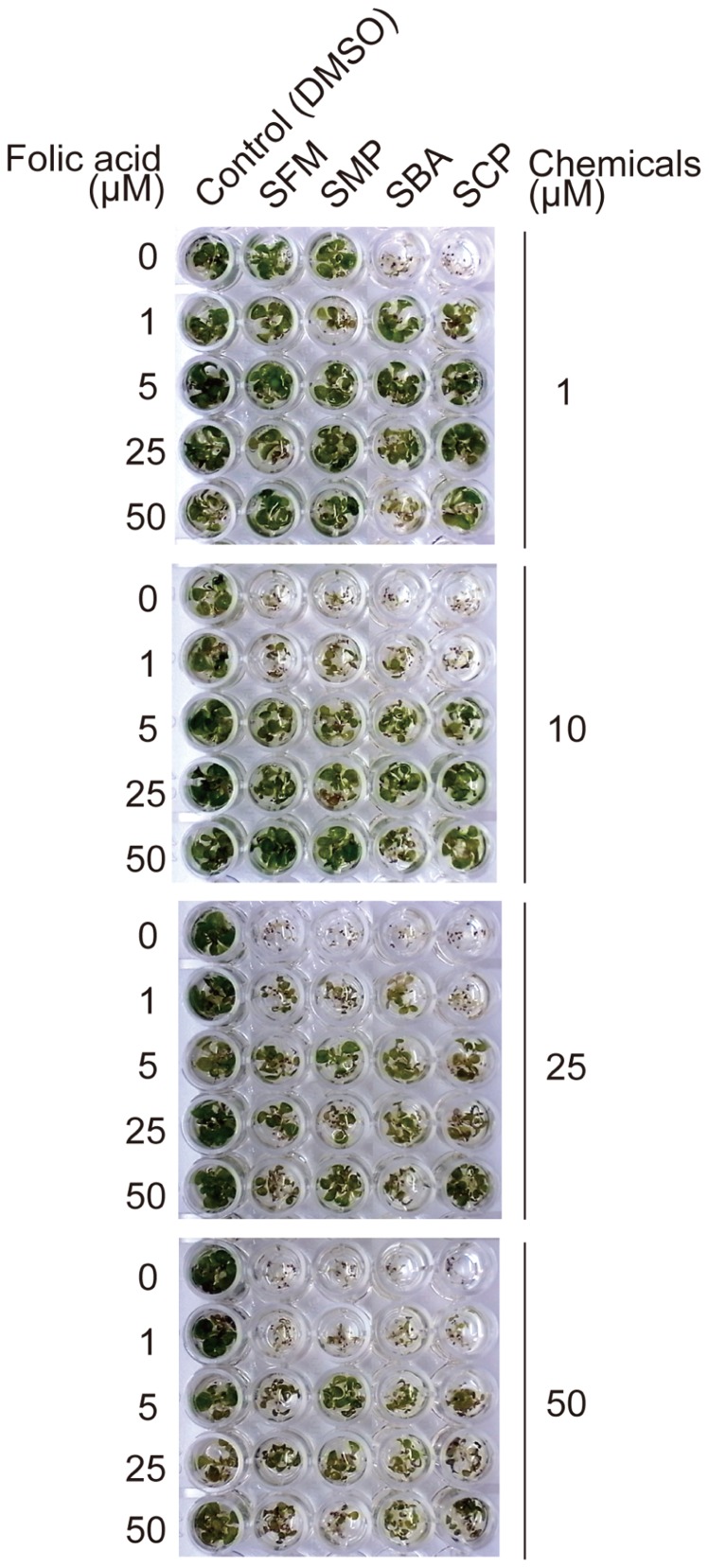
**The effect of the sulfonamides on germination of *Arabidopsis thaliana***. *Arabidopsis* seeds were dispensed into each well of a 96-well plate, and liquid media containing each sulfonamide and folic acid at the indicated concentrations were applied, followed by incubation under long-day conditions at 22°C. Photographs were taken after 2 weeks. These data are representative of two independent replicates with similar results.

**FIGURE 9:**
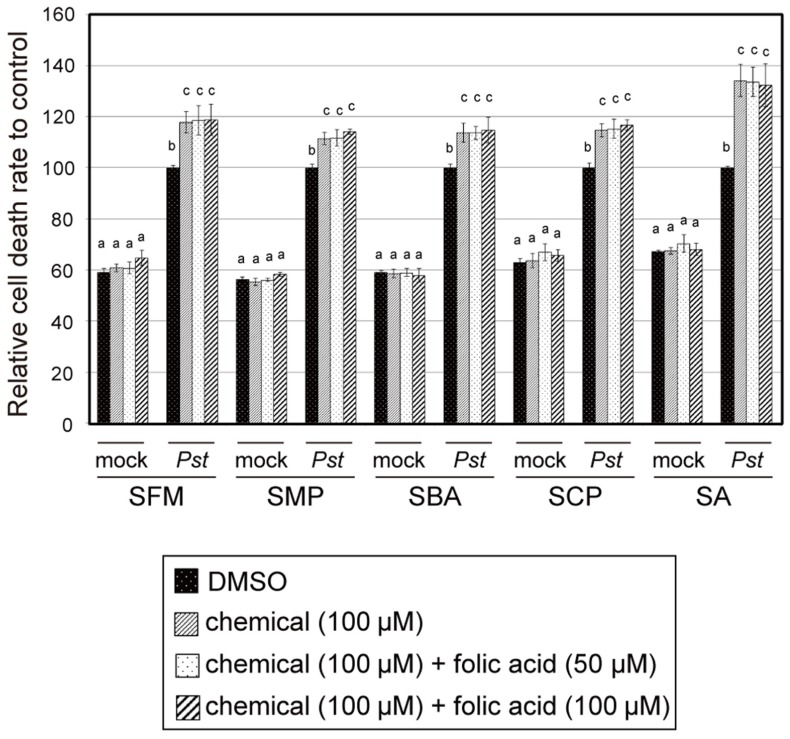
**The effect of folate on the sulfonamide-dependent cell death enhancement of *Arabidopsis* suspension cultures induced by *Pst-avrRpm1***. The sulfonamide compounds and folate were incubated with *Arabidopsis* suspension-cultured cells at the indicated concentrations, with or without the avirulent bacterial pathogen *Pst-avrRpm1*, and the degree of cell death was measured in terms of the concentration of Evans blue dye. Each cell death rate is shown as a value relative to the mean of the mock pathogen-treated group. Sodium salicylate (SA) was used as a positive control. The error bars represent the mean ± SE values of five independent replicates. The letters indicate statistically significant differences between treatments (one-way ANOVA, Tukey’s *post hoc* test, *P* < 0.05). The experiment was repeated three times with similar results.

To determine the relationship between folate biosynthesis inhibition and the plant immune-priming effect of the sulfonamides, we examined the effect of the isolated sulfonamides on plant growth. After sterilization and stratification, *Arabidopsis* seeds were incubated with MS liquid media containing sulfonamides at varied concentrations, as shown in **Figure 8**. Growth of *Arabidopsis* seedlings was severely suppressed by the sulfonamides (**Figure 8**). SBA and SCP prevented growth at 1 μM, whereas SFM and SMP inhibited growth at 10 μM. The growth-suppression effects of these compounds were restored by the addition of folate in a concentration-dependent manner (**Figure 8**). These results indicate that the isolated sulfonamides inhibit the growth of *Arabidopsis* seedlings through the perturbation of folate biosynthesis. However, the sulfonamides did not exhibit potentiation effects on pathogen-induced cell death at concentrations lower than 10 μM. This means that the inhibitory effect of the sulfonamides on folate biosynthesis would be independent of its chemical property for immune-priming. To confirm this hypothesis, we examined if application of folate can suppress the effect of the sulfonamides on pathogen-induced cell death. As shown in **Figure 9**, the enhancement of pathogen-induced cell death by the four sulfonamides was not suppressed by the addition of at least 100 or 50 μM of folate. These same concentrations of folate restore the seedling growth-suppressive effects of the sulfonamides. Therefore, these data suggest that different mechanisms of action are responsible for sulfonamide effects on seedling growth and pathogen-induced cell death.

## DISCUSSION

In this study, we screened two different chemical libraries by using the established high-throughput chemical screening method for plant immune-priming compounds (Noutoshi et al., 2012a). Both chemical libraries are composed of bioactive substances and natural products, and the sulfonamide compounds were independently identified from each library (**Figure 1**). Although these compounds were obtained from a screening by using suspension cells, they conferred disease resistance to *Arabidopsis* whole plants against both virulent and avirulent strains of pathogenic bacteria (**Figure 3**). These results demonstrate that our screening procedure can be used for the isolation of lead plant activators as we expected.

These sulfonamides did not affect bacterial growth at the effective concentration range for the priming of immunity (**Figure 7**). However, they prevented *Arabidopsis* germination through the inhibition of folate biosynthesis (**Figure 8**). Our result is consistent with those of previous reports in that sulfanilamide, sulfadiazine (Sdiz), and sulfacetamide inhibit the enzymatic activity of dihydropteroate synthase (DHPS) *in vitro* with estimated IC_50_ values of 18.6, 9.6, and 4.2 μM, respectively (Prabhu et al., 1997) and Sdiz prevents *Arabidopsis* germination at 1 mg/L (4 μM; Hadi et al., 2002). However, at least overnight incubation with the sulfonamides at 100 μM did not affect the viability of the suspension cells (**Figure 2**) and thus they were isolated by our screening. This is consistent with the report that lethal doses of other sulfonamide compounds, sulfamethoxazole (Smex), Sdiz, and sulfapyridine (Spyr), for *Arabidopsis* seedlings are 2 mM or more (Schreiber et al., 2008). These reports indicate that *Arabidopsis* is sensitive to sulfonamides only at early developmental stages, which could be due to the presence of less folic acid for *de novo* DNA synthesis. To avoid phytotoxic activity, the sulfonamides would need to be applied conditionally for immune activation.

As described above, sulfonamides are known to mimic PABA and inhibit folate biosynthesis (Brown, 1962). Because PABA is biosynthesized from chorismate in the shikimate pathway, sulfonamide application might increase the cellular chorismate pool. A large amount of SA is synthesized *de novo* during disease resistance responses by the function of isochorismate synthase (Wildermuth et al., 2001); therefore, the sulfonamides might facilitate SA accumulation in response to pathogen challenge. Based on the analogy that the sulfonamides can mimic a benzoate derivative PABA, we speculated that the sulfonamides might be able to mimic SA at a high concentration range. However, the sulfonamides did not behave like SA at least at 100 μM (**Figure 5**). We have previously identified imprimatin A and B as plant immune-priming compounds from the same screening system and found that they enhanced immune responses by inhibiting the SAGTs involved in SA metabolism (Noutoshi et al., 2012a,b). These imprimatin compounds were incorporated into SAGTs instead of SA and inhibited the glucosylation of SA (Noutoshi et al., 2012a). As demonstrated in **Figure 6**, the sulfonamides did not function as SAGT inhibitors; therefore, they behave differently from imprimatin A and B.

Recently, sulfamethazine (SMZ) was isolated as a chemical suppressor of epigenetic silencing (Zhang et al., 2012). SMZ treatment suppresses the silencing of transgenes, endogenous transposons, and other repetitive elements. These chemical effects are due to decreased cellular pools of folate and *S*-adenosylmethionine and leads to a reduction in DNA methylation levels and histone H3 Lys-9 dimethylation levels (Zhang et al., 2012). The involvement of epigenetics in priming events was also suggested by another report (Jaskiewicz et al., 2011). The increased levels of methylation and acetylation of lysine residues of the histones H3 and H4 were observed on the promoter regions of the *WRKY6*, *WRKY29*, and *WRKY53* genes in primed plants after treatment with BTH or a pathogen (Jaskiewicz et al., 2011). Although the methylation status in these reports is not generally consistent, these findings led us to speculate that alteration of histone modifications induced by folate biosynthesis inhibition could be a cause of the immune-priming of the sulfonamide compounds. However, since application of folate did not restore the sulfonamide-induced cell death enhancement (**Figure 9**), we conclude that the sulfonamides upregulate the pathogen-induced cell death through an unknown mechanism.

Sulfonamide compounds were also identified as a protectant for *A. thaliana* whole seedlings exposed to bleaching induced by the cocultivation of virulent *Pst* (Schreiber et al., 2008). Our results showed that 100 μM of Smex significantly suppressed the growth of bacterial pathogens inside *Arabidopsis* seedlings. Smex has also been found to suppress symptoms of infection by a cereal pathogen *Fusarium graminearum* in both *Arabidopsis* and wheat (Schreiber et al., 2011). Interestingly, sulfanilamide has no plant-protective activity although it has a structure closely similar to that of the isolated sulfonamides (Schreiber et al., 2008, 2011). Recently, we have also identified three diuretic compounds, bumetanide, bendroflumethiazide, and clopamide, as plant immune-priming compounds from the screening (Noutoshi et al., 2012c). Interestingly, these three diuretics share the same sulfonamide functional group (Noutoshi et al., 2012c). Considering these findings, only RSO_2_NH_2_ with particular R-groups might have immune-activating properties in plants. Large-scale structure–activity relationship analysis using various sulfonamide derivatives will help identify minimal structural requirements for immune activity as well as provide effective leads for practical application.

## Conflict of Interest Statement

The authors declare that the research was conducted in the absence of any commercial or financial relationships that could be construed as a potential conflict of interest.
